# Involvement of Frontal Functions in Pain Tolerance in Aging: Evidence From Neuropsychological Assessments and Gamma-Band Oscillations

**DOI:** 10.3389/fnagi.2020.00131

**Published:** 2020-05-27

**Authors:** Shu Zhou, Ségolène Lithfous, Olivier Després, Thierry Pebayle, Xiaoying Bi, André Dufour

**Affiliations:** ^1^Laboratoire de Neurosciences Cognitives et Adaptatives, UMR 7364 – Université de Strasbourg – CNRS, Strasbourg, France; ^2^Department of Neurology, Changhai Hospital, Second Military Medical University, Shanghai, China; ^3^Centre d’Investigations Neurocognitives et Neurophysiologiques, UMS 3489 – Université de Strasbourg – CNRS, Strasbourg, France

**Keywords:** tonic pain, frontal cortex, executive functions, gamma-band oscillations, normal aging

## Abstract

Reduced pain tolerance may be one of the possible explanations for high prevalence of chronic pain among older people. We hypothesized that age-related alterations in pain tolerance are associated with functioning deterioration of the frontal cortex during normal aging. Twenty-one young and 41 elderly healthy participants underwent a tonic heat pain test, during which cerebral activity was recorded using electroencephalography (EEG). Elderly participants were divided into two subgroups according to their scores on executive tests, high performers (HPs; *n* = 21) and low performers (LPs; *n* = 20). Pain measures [exposure times (ETs) and perceived pain ratings] and cerebral activity were compared among the three groups. ETs were significantly lower in elderly LPs than in young participants and elderly HPs. Electroencephalographic analyses showed that gamma-band oscillations (GBOs) were significantly increased in pain state for all subjects, especially in the frontal sites. Source analysis showed that GBO increase in elderly LPs was contributed not only by frontal but also by central, parietal, and occipital regions. These findings suggest that better preservation of frontal functions may result in better pain tolerance by elderly subjects.

## Introduction

Elderly individuals tend to more frequently experience chronic pain than younger adults (Gibson and Farrell, 2004; Lautenbacher, 2012). The decreased tolerance of sustained pain by elderly individuals could be one of the reasons. Although age-related alterations in the peripheral pathway (mainly Aδ fibers) may result in the insensitivity of pain (Kemp et al., 2014), accumulative evidences have indicated a vital contribution of central mechanism in geriatric pain.

The frontal cortex may play an important role in pain processing. Studies have shown that pain-related experimental stimuli (i.e., sustained/tonic pain, sensitization to pain) elicited activity within the prefrontal cortex (PFC), involved in the subjective perception of pain (Schulz et al., 2015) and pain modulation (Lorenz et al., 2003; Seifert et al., 2009). The orbitofrontal cortex has shown functional connectivity with the periaqueductal gray that is known to play a crucial role in descending inhibition of nociceptive inputs (Valet et al., 2004; Kong et al., 2010). The frontal cortex also sustains executive functions, including inhibition, working memory, flexibility, and higher mental processes such as planning and problem solving (Alvarez and Emory, 2006; Chan et al., 2008). Executive functions may influence the ability of an individual to tolerate pain. For example, inhibition (Oosterman et al., 2010) and working memory (Buhle and Wager, 2010; Legrain et al., 2013) abilities have been associated with better pain tolerance and modulation in younger subjects, suggesting an important relationship between tolerance of sustained pain and executive functions.

During aging, gray matter losses in the frontal cortex are greater than in most other areas of the brain (Raz et al., 2007; Pfefferbaum et al., 2013). Executive functions have shown age-associated reductions (Verhaeghen and Cerella, 2002) generally with considerable interindividual variations (Moy et al., 2011). However, during executive function tasks, older adults seem to increasingly recruit the frontal network to compensate for age-related functional declines (Turner and Spreng, 2012). Nevertheless, if cognitive load of the task exceeds compensatory capacity, performance declines in concert with decreasing activation (Daffner et al., 2011; Sebastian et al., 2013). Concerning to pain processing, our previous works together with the others’ investigations have shown that, in the elderly subjects, reduced tolerance and cognitive modulation of tonic pain were associated with declines in executive functions (Marouf et al., 2014; Zhou et al., 2015a, b). Recently, based on a larger sample database and complete neuropsychological assessments, we further observed that reductions in executive functions subtended by the frontal network are associated with the lack of descending inhibitor control of pain in healthy elderly participants (Lithfous et al., 2019). Nevertheless, the cerebral activities during tolerance of sustained pain in the healthy elderly adults remain undetermined to directly confirm the involvement of frontal function.

This study therefore investigated the role of frontal functions in pain tolerance during aging. Healthy young and elderly participants underwent tonic pain tests, during which cerebral activity was recorded by electroencephalography (EEG). Given the lack of exactly pain-related brain signature (Mouraux and Iannetti, 2018), we chose to focus on gamma-band oscillations (GBOs) (40–100 Hz). Study in rodents recently showed that gamma-band event-related synchronization was the only response that reliably correlated with pain-related behavior (Peng et al., 2018). Human intracranial EEG investigations also observed that the enhancement of GBOs was preferential in response to nociceptive stimuli compared to salience-controlled non-nociceptive stimuli. Thus, GBOs might be a “pain-selective” brain activity (Hu and Iannetti, 2019). When elicited by transient nociceptive stimuli, this brain activity was present particularly in insular sites, less prominent, but as well as in temporal and frontal regions (Liberati et al., 2018a, b). When related to tonic pain processing, GBOs have been observed in the PFC, probably representing the summary effects of bottom-up stimulus-related and top-down subject-driven cognitive processes (Peng et al., 2014; Schulz et al., 2015). Moreover, GBOs have also been implicated in somatosensory and cognitive processing (Bertrand and Tallon-Baudry, 2000; Goffaux et al., 2004; Kaiser and Lutzenberger, 2005; Jensen et al., 2007). Therefore, EEG-based analysis on GBOs was used to explore the involvement of the frontal cortex in aging-related pain processing.

## Materials and Methods

Healthy young and elderly participants underwent tonic pain tests. Elderly subjects were selected from a database of 400 elderly (aged >60 years) healthy adults. In order to maximize statistical power, we divided elderly participants into two subgroups according to their scores on neuropsychological tests examining executive functions (i.e., high and low performers). Pain measures [i.e., exposure times (ETs) and pain judgments] and cerebral activity recorded by EEG were compared among the three groups of subjects.

### Participants

Elderly subjects were selected from a database of 400 elderly (aged >60 years) healthy adults. All elderly subjects of this database were recruited by means of advertisements from December 2013 to January 2017 and underwent complete neuropsychological assessments, including global efficiency, episodic memory, visuospatial abilities, language, executive functions, and mental disorders, such as anxiety and depression. Subjects with Mini Mental State Examination scores <25 (Folstein et al., 1975) and subjects with depressive/anxiety disorders, defined as a Geriatric Depression Scale score >5 (Yesavage et al., 1982) and a State-Trait Anxiety Inventory (STAI) mean score >55 (Kvaal et al., 2005), were excluded. Based on their performance on several executive functions tests (see section “Neuropsychological Examinations” for details), elderly participants were selected and divided into two subgroups, high performers (HPs) and low performers (LPs), maximizing the variability of executive functions in elderly participants, thus increasing the statistical power (see below for a detailed explanation of the selection method). Younger adults were recruited through advertisement in the local newspapers.

All study participants underwent medical examinations to exclude conditions that could alter pain perception: (1) cutaneous lesions, peripheral neuropathy, multiple sclerosis, diabetes, brain lesion history (e.g., stroke, trauma, etc.), and psychiatric disorders; (2) chronic pain conditions, defined as daily pain for 3 months or more during the previous year; and (3) subjects using analgesic or psychotropic medications at the time of the study. Fear and anxiety associated with pain and subjective experience of pain were assessed in each participant using the Fear of Pain Questionnaire (FPQ) (Asmundson et al., 2008) and the Pain Catastrophizing Scale (PCS) (French et al., 2005), respectively. All participants were paid and provided written informed consent prior to participation. All procedures were approved by the local ethics committee and were in line with the principles of the Declaration of Helsinki.

A total of 62 subjects were recruited (Table 1), including 21 young adults (10 males, 11 females; mean age = 23.0 ± 3.6 years), 21 elderly HPs (10 males, 11 females; mean age = 68.5 ± 5.6 years), and 20 elderly LPs (10 males, 10 females; mean age = 68.5 ± 6.2 years). Participants of all three groups were matched with regard to the level of education. Elderly participants in each group were also matched on the time period between their complete neuropsychological assessment and their participation to the present study (i.e., 6–8 months).

**TABLE 1:** Demographic characteristics and performances on executive functions tests.

	**Young (*n* = 21)**		**Old HPs (*n* = 21)**		**Old LPs (*n* = 20)**		
	**Mean**	**SD**	**Mean**	**SD**	**Mean**	**SD**	***p***
**Demographic data**							
Age (years)	22.95	3.58	68.52	5.56	68.45	6.24	
Sex (women/men)	11/10	–	11/10	–	10/10	–	
MMSE	–	–	29.14	0.65	27.45	1.61	<0.001
Anxiety-state (STAI-S)	30.90	7.92	26.53	4.57	26.94	5.36	0.063
Anxiety-trait (STAI-T)	40.67	7.19	39.82	6.00	31.17	5.60	<0.001
Depression (BDI/GDS)	3.67	4.73	2.12	1.65	1.33	1.41	0.07
FPQ	57.86	11.85	48.25	15.11	41.16	12.46	<0.001
PSC	19.00	10.45	16.28	11.55	9.95	9.11	0.026
**Executive functions**							
FAB (*Z* score)	–	–	0.68	0.15	−1.33	0.11	<0.001
DSB (*Z* score)	–	–	−0.42	0.42	−0.83	0.57	0.049
Letter fluency (*Z* score)	–	–	0.75	1.06	−1.10	1.12	<0.001
TMT B-A (*Z* score)	–	–	0.73	0.16	−0.21	0.53	<0.001
Stroop (*Z* score)	–	–	0.53	0.41	−0.04	0.51	0.052
**Frontal composite score**	–	–	0.45	0.31	−0.70	0.51	<0.001

*SD, Standard Deviation; MMSE, Mini Mental State Examination; STAI, State-Trait Anxiety Inventory; BDI, Beck Depression Inventory for younger participants; GDS, Geriatric Depression Scale for older participants; FPQ, Fear of Pain Questionnaire; PCS, Pain Catastrophizing Scale. FAB, Frontal Assessment Battery; DSB, Digit Span Backward; TMT, Trail Making Test.*

### Neuropsychological Examinations

Executive functions were evaluated using the following tests: (1) the Frontal Assessment Battery (Dubois et al., 2000), evaluating general executive functions on six subtests: abstract reasoning (similarities), mental flexibility (phonological verbal fluency), motor programming (Luria’s motor series), interference (conflicting instructions), inhibitory control (go–no-go task), and environmental autonomy (grasping); (2) the Digit Span Backward test (Ramsay and Reynolds, 1995), evaluating working memory; (3) phonemic fluency, evaluating mental flexibility; (4) the Trail Making Test, evaluating attention and task switching; and (5) the Stroop test (Godefroy, 2007), evaluating inhibition. The use of multiple tests to assess executive functions was regarded as more sensitive than a single test in detecting mild executive deficits in elderly participants.

All scores were compared to the general population norms and standardized relative to age and education matched norms converted into *Z* scores. All participants included in this study had normal scores on neuropsychological assessments (i.e., above the −1.96 *Z* score threshold, which is a criterion of dementia in clinical examinations) and were therefore cognitively intact. Elderly participants were allocated to either LP or HP group according to their performance on these examinations (Table 1). The LP subgroup consisted of elderly subjects with scores below a “supra-pathological” cutoff line (i.e., *Z* scores < −0.68) on at least two neuropsychological tests, and the elderly HP subgroup with frontal composite scores above. This cutoff line was set above the criteria of mild cognitive impairment (*Z* scores < −1.5) in order to increase the sensitivity of detecting slighter deficits in executive functions during normal aging (Lithfous et al., 2018). We calculated the mean *Z* scores for all the five tests of executive functions, defined as the frontal composite score, to provide a globe index evaluation for frontal function. The goal of this study was to observe the individual variability within elderly population; therefore, the young group was recruited as a control group for default optimal performance on neuropsychological assessments.

### Pain Tolerance Test

#### Procedure

**FIGURE 1:**
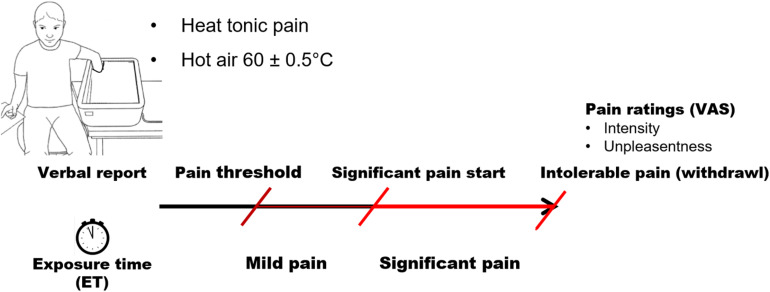
Paradigm of tonic pain procedure.

Pain tolerance was determined using a tonic heat pain model (Figure 1). Pain was induced by exposing each participant’s hand to hot air at 60.0°C ± 0.5°C. Hot air was produced by Peltier modules and circulated in a hermetic box made by our laboratory. This model at 70.0°C can produce intensive pain perception within 3 min after the pain threshold (Zhou et al., 2015a); we therefore choose a lower temperature to extend ET during pain perception. The heat pain model shows less confounding with thermoregulatory cardiovascular reactivity observed in tonic cold pain model (Naert et al., 2008).

Participants were instructed to insert their entire left hand into the box and keep it open and still. All participants were right-handed and, consequently, were able to score precisely their pain perception on the visual analog scale (VAS) with their dominant hand after withdrawal of the left (non-dominant) hand. Hand temperature was monitored continuously throughout the entire procedure. Subjects were required to announce three stages of sensation: (1) the first pain sensation (pain threshold); (2) significant pain, that is, when pain was rated 50 on a 100-point VAS; and (3) when pain became intolerable. The test ended when each participant took his/her hand off the box or 10 min after the pain threshold was reached. This maximum ET was set because our preliminary tests of the model showed that an air temperature of 60°C induces a mean maximum hand temperature of 42°C ± 1°C in 8 ± 2 min, followed by a consistent hand temperature with small fluctuations within 1°C. Perception of pain accordingly remained unchanged or was even reduced in a few subjects.

Pain tolerance was estimated by the ET to pain. Three types of ET were measured (Figure 1): ET-mild pain, defined as the time from the pain threshold to significant pain; ET-significant pain, defined as the time from the announcement of significant pain to the withdrawal of the hand; and ET-total, defined as the time between the pain threshold and the removal of the hand. Perceived pain intensity and unpleasantness were assessed using VAS. Participants rated pain intensity from no pain (0) to worst possible pain (100) and unpleasantness from not unpleasant (0) to extremely unpleasant (100).

#### Electrophysiological Recording and Analysis

**FIGURE 2:**
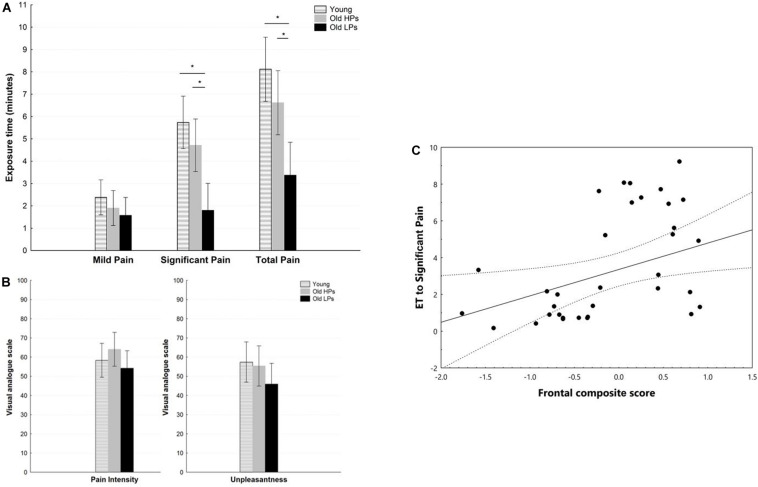
Pain measures during pain tolerance tests. **(A)** Exposure time, **(B)** perceived pain ratings for pain intensity and unpleasantness, **(C)** correlation between frontal composite score and exposure time to significant pain. *p < 0.05 for *post hoc* analyses of paired comparisons.

Electroencephalograms were recorded during 2-min eyes-open resting state (i.e., pain-free state) and subsequently the tonic pain test (for details, see section “Procedure”) from 32 Ag/AgCl active electrodes (BioSemi^®^; Amsterdam, Netherlands) mounted in an elastic cap. Electrode positions included the standard International 10–20 system location and intermediate positions (Klem et al., 1999). The EEG was digitized at 512 Hz, with an amplified band-pass of 0.1–100 Hz. Additional electrodes were placed on earlobes as references (averaged offline). Vertical and horizontal electro-oculographic (EOG) potentials were recorded from bipolar derivations to detect ocular movements. In order to minimize noise originated from movement, participants were instructed to fixate a black computer screen placed in front of them, keep still, and not talk except for verbal announcing three stages of sensation (i.e., “pain started,” “significant pain started,” and “intolerable pain”). Pain ratings were assessed after the tonic pain test (i.e., after withdrawal of the hand), to avoid motor artifacts of ongoing EEG recording during the test. To focus on brain responses related to pain processing, GBO data of pain were limited to the ET-significant pain period (varying from 1.1 min to 8.6 min, on average 4.3 min; Figure 2) to ensure that brain activities of pain processing were represented.

The EEG data were preprocessed using EEGLAB, an open source toolbox running in the MATLAB environment (Delorme and Makeig, 2004), and in-house MATLAB functions. Data preprocessing consisted of the following steps: (1) the recorded EEG signals were band-pass filtered between 0.5 and 100 Hz; (2) a notch filter was used to eliminate 50-Hz line noise; (3) EEG data were re-referenced to a common average reference; (4) data portions with large drift were removed; (5) channels with bad activation were interpolated using a spherical spline method; (6) EEG epochs were then visually inspected, and trials contaminated by artifacts due to saccadic movement detected by EOG were removed; (7) the Blind Source Separation was used to correct the data portions contaminated by eye blinks and movements, electromyography, or any other non-physiological artifacts; (8) the data portions reflecting resting-state period (RS) and significant pain period (SP) were extracted, respectively; (9) the EEG signals within the above two periods were segmented into 1,000-ms epochs; (10) EEG epochs with amplitude values exceeding ±80 μV at any electrode were rejected. For a SP, an average of 79 epochs, 199 epochs, and 318 epochs (71.1%, 68.3%, and 88.1% of the total number of epochs) were remained for elderly LPs, elderly HPs, and young participants, respectively.

The EEG spectral power was analyzed with in-house MATLAB functions. For each participant and each period, the segmented epochs were transformed to the frequency domain based on fast Fourier transforms (FFTs), yielding FFTs ranging from 1 to 100 Hz with frequency resolution 1 Hz. The power spectra were calculated as the magnitude-squared FFTs averaged across epochs. The EEG power of each frequency band was obtained for each electrode, each period, and each participant through averaging the power spectral of each frequency bin within its corresponding frequency limits: delta (∼1–4 Hz), theta (∼4–8 Hz), alpha (∼8–13 Hz), beta (∼14–30 Hz), low gamma (∼30–48 Hz), and high gamma (∼52–100 Hz). We focused on high-frequency brain oscillations in the gamma band (52–100 Hz). We computed the pain-related change of powers between the two periods (i.e., the RS and SP) for each electrode and each participant, using the following equation: *pain-related change of powers%* = *(SP - RS)/RS*, where SP is the spectral power within the SP, and RS is the spectral power within the RS. The SP was chosen to ensure the recorded oscillations were induced by tolerating pain.

Artifact-free EEGs were quantitatively analyzed to determine source localization using standardized low-resolution brain electromagnetic tomography (sLORETA), determined using LORETA-KEY^©®^, a publicly free academic software, located at http://www.uzh.ch/keyinst/loreta.htm (Pascual-Marqui et al., 2011). Among all the source localization methods, the sLORETA provides a weighted minimum norm inverse solution and has correct localization even in the presence of structured noise, albeit with low spatial resolution (Aoki et al., 2015). The solution space of sLORETA is restricted to cortical and divided into 6,239 cortical gray matter voxels at 5-mm resolution using the MNI152 template. The amplitude of each EEG rhythm (i.e., delta, theta, alpha, beta, and gamma) was obtained for each participant and each period (i.e., RS and SP). The source signals were analyzed through the following two approaches. In the first approach, we explored the cortical distribution of group-level source solutions of GBOs during SP state. The 6,239 cortical gray matter voxels were divided into 84 Brodmann areas. Group-level source solutions were obtained via averaging the source solutions across subjects in each group. The estimated localization of GBOs during SP was identified as the 10 Brodmann areas with largest amplitudes. In the second approach, changes in GBOs induced by pain were estimated by one-sample *t* test conducted on the pain-related change of GBOs (i.e., comparing SP from RS) for each group and each voxel. The localization of GBO changes was resulted in three-dimensional images where cortical voxels of statistically significant differences were identified. The significance level (*p* value) was corrected using false discovery rate procedure for multiple comparisons.

### Statistical Analysis

Statistical tests were carried out using STATISTICA (StatSoft, Inc., version 8.0^1^). The normality of data distribution was determined using the Kolmogorov–Smirnov test, with statistical significance set at *p* < 0.05.

To investigate the relationship between executive functions and pain tolerance abilities, group comparisons were performed with measures of pain tolerance as the within-subject factor and group (i.e., young subjects and elderly HPs and LPs) as a between-subject factor. Inasmuch as ETs showed a non-parametric distribution, Kruskal–Wallis analysis of variance (ANOVA) was used to compare ETs across the three groups, and Kruskal–Wallis multiple comparisons were used as *post hoc* tests when group had a significant effect. One-way ANOVA was used for group comparisons of normally distributed pain ratings. Newman–Keuls *post hoc* analyses were used when ANOVA showed significant effects. Potential pain-related confounders (i.e., anxiety, depression, FPQ, and PCS) showing between-group difference were identified as the covariants of pain measurements and statistically controlled by analysis of covariance (ANCOVA). Pearson correlations were assessed between frontal composite score and pain tolerance measurement.

For pain-elicited spectral power comparisons, averaged spectral power within each frequency band at frontal (AF3, AF4, Fz), central (CP1, CP2, Cz), parietal (P7, P8, Pz), and temporal (T7, T8, CP5, CP6) electrodes was calculated for each subject and two states (i.e., pain free and significant pain). First, two-way ANOVA was undertaken for all frequencies on electrodes of different regions to examine the main effects for “group” (young, elderly HP, and LP) and “state” (pain free and significant pain), as well as the interaction between the two factors. Second, when the state effect was significant, two-way ANOVA was performed with factors of “ROI” and “group” on the normalized pain-related change of brain activities [i.e., (pain - rest)/rest] to test the scalp distribution of pain-related changes of power. When the main effect of the ANOVA was significant, Newman–Keuls *post hoc* tests were performed. Pearson correlations were assessed between pain-related changes of power and pain tolerance measurement. Bonferroni corrections to the significance level have been made for factors with more than 2 modalities.

## Results

Twenty-one young adults (10 males, 11 females; mean age = 23.0 ± 3.6 years), 21 elderly HPs (10 males, 11 females; mean age = 68.5 ± 5.6 years), and 20 elderly LPs (10 males, 10 females; mean age = 68.5 ± 6.2 years) (Table 1) underwent tonic pain tests.

### Between-Group Comparison of Pain Tolerance Measures

Statistical analyses revealed a significant effect of group on ET to significant pain [*H*_(__2_,_5__9__)_ = 17.77, *p* < 0.001] and total ET [*H*_(__2_,_5__9__)_ = 14.86, *p* < 0.001]. *Post hoc* analyses showed that ET to significant pain was significantly shorter in elderly LPs than in young participants (*p* < 0.001) and elderly HPs (*p* = 0.008). Similar results were observed for total ET, which was significantly shorter in elderly LPs than in young participants (*p* < 0.001) and elderly NPs (*p* = 0.025) (Figure 2A). In contrast, ratings of perceived pain and unpleasantness did not differ significantly among the three groups, *F*_(__2_,_59__)_ = 1.26, *p* = 0.290; *F*_(__2_,_59__)_ = 1.3319, *p* = 0.273, respectively (Figure 2B). Correlation analyses showed that lower frontal composite score was associated with shorter total ET (*r* = 0.369, *p* < 0.001) and ET to significant pain (*r* = 0.377, *p* < 0.001, Figure 2C).

Affective statement and attitude toward pain may impact pain tolerance. Comparisons of demographic characteristic showed that elderly participants, especially elderly LPs, had lower ratings of anxiety (STAI-trait: *F* = 12.67, *p* < 0.01), fear of pain (FPQ: *F* = 8.08, *p* < 0.01), and catastrophizing of pain (PCS: *F* = 3.93, *p* = 0.03) than the younger participants. Consequently, STAI-state, FPQ, and PCS ratings were prepared to be included as a between-group covariant of pain tolerance measurement (i.e., pain rating and ET) in ANCOVAs and statistically controlled. Results revealed that group effect remained significantly different for total ET (*p* < 0.01) and ET to significant pain (*p* = 0.017) after controlling the level of anxiety, fear, and catastrophizing of pain. Ratings of perceived pain and unpleasantness did not differ significantly among the three groups after controlling the above covariants.

### Power Spectral Comparisons for All Frequency Bands

**FIGURE 3:**
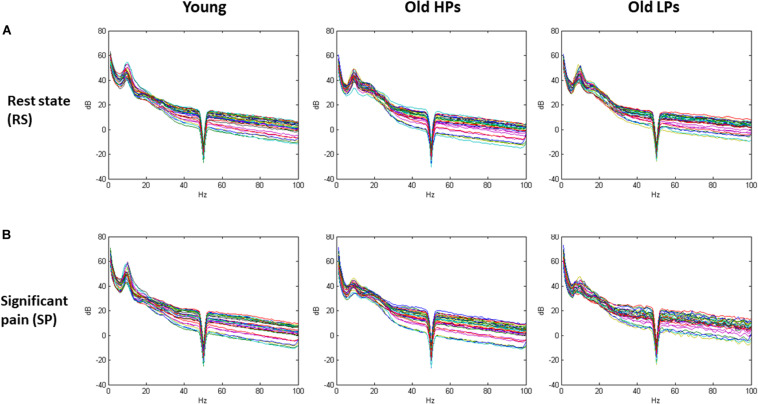
Spectral waveform in the pain-free and significant pain states for each group. **(A)** Spectral waveform during resting-state and **(B)** significant pain state.

Spectral waveform in the pain-free and significant pain states for each group is displayed in Figure 3.

**FIGURE 4:**
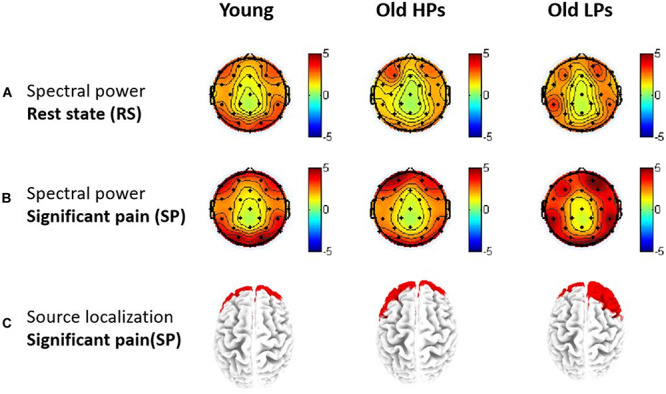
Topographies of gamma-band oscillations (GBOs). **(A)** Spectral power maps during resting-state and **(B)** significant tonic heat pain. **(C)** Source localization of areas with largest GBO power during significant tonic heat pain.

Two-way ANOVA of group × state effect revealed significant group effect within theta frequency (frontal, central, and parietal electrodes) and gamma frequency (parietal electrodes). Significant state effect was observed within both low and high gamma frequency at frontal electrodes (low gamma *F* = 9.59, *p* = 0.007; high gamma *F* = 7.515, *p* = 0.003) (Table 2 and Figure 4). However, no significant difference was found on group × state interaction. *Post hoc* analysis on group effect revealed that, within theta frequency, younger participants showed higher theta power than both elderly HPs and elderly LPs at frontal (young vs. elderly HPs, *p* = 0.006; young vs. elderly LPs, *p* = 0.018), central (Young vs. elderly HPs, *p* < 0.001; young vs. elderly LPs, *p* = 0.004), and parietal (young vs. elderly HPs, *p* = 0.004; young vs. elderly LPs, *p* = 0.004) electrodes, whereas no significant difference was observed between the two elderly groups. Similar results were observed within low gamma frequency at parietal electrodes, showing increased low gamma power than both elderly HPs and elderly LPs (young vs. elderly HPs, *p* = 0.007; young vs. elderly LPs, *p* = 0.019).

**TABLE 2:** Two-way ANOVA (group × state) comparisons of spectral power for all frequency bands.

	**Group effect**	**State effect**	**Group** × **state effect**
**Theta**			
Frontal	0.005*	0.148	0.80
Central	<0.001*	0.708	0.947
Parietal	0.002*	0.470	0.980
Temporal	0.336	0.638	0.916
**Alpha**			
Frontal	0.328	0.947	0.966
Central	0.185	0.720	0.901
Parietal	0.186	0.961	0.910
Temporal	0.843	0.868	0.650
**Beta**			
Frontal	0.94	0.633	0.790
Central	0.318	0.983	0.797
Parietal	0.596	0.721	0.747
Temporal	0.218	0.771	0.731
**Low-gamma**			
Frontal	0.697	0.007*	0.885
Central	0.030	0.241	0.981
Parietal	0.007*	0.076	0.881
Temporal	0.344	0.030	0.953
**High-gamma**			
Frontal	0.273	0.003*	0.492
Central	0.023	0.302	0.288
Parietal	0.758	0.132	0.251
Temporal	0.168	0.072	0.417

*Significance difference after Bonferroni correction for multiple comparisons.

Source location analysis also revealed that GBOs induced by significant pain state were mainly located in the frontal region in three groups (Figure 4C). Precisely, the 10 Brodmann areas with largest amplitudes in gamma activity were 10R, 11R, 11L, 10L, 46R, 9R, 38R, 47R, 8R, and 17R for elderly LPs; 10L, 11L, 11R, 10R, 38L, 47L, 46L, 9L, 25L, and 32L for elderly HPs; and 11L, 10L, 11R, 10R, 47L, 38L, 46L, 47R, 38R, and 25L for younger participants, respectively.

### Pain-Related Change of GBOs

**FIGURE 5:**
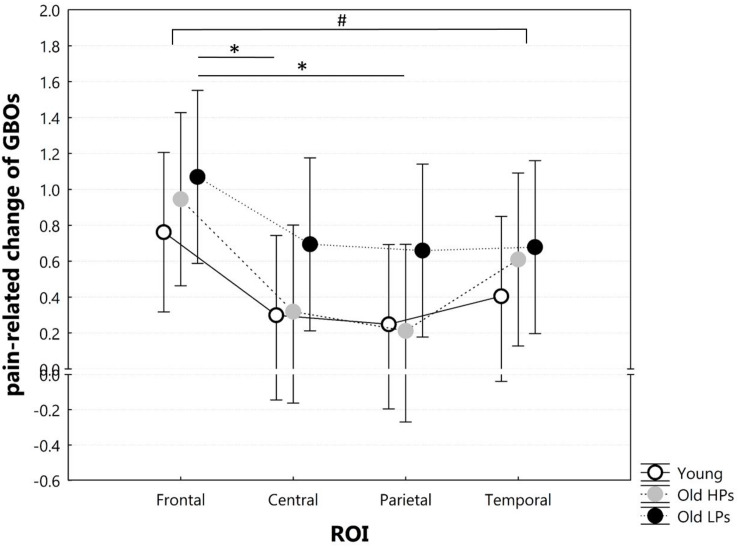
Two-way ANOVA of group × ROI on pain-related change of GBOs. ROI defined as frontal (AF3, AF4, Fz), central (CP1, CP2, Cz), parietal (P3, P4, Pz), temporal (T7, T8, CP5, CP6). ^#^Significant ROI effect on pain-related change of GBOs. *Significant difference for *post hoc* tests after Bonferroni correction for multiple comparisons.

Above analysis showed pain-related change (i.e., state effect) of brain activities was mainly observed within gamma frequency; thus, we focused on power change of GBOs. Spectral power of GBOs in both states is presented in Figure 5.

Two-way ANOVA of group × ROI on pain-related change of GBOs showed significant ROI effect (*F* = 7.536, *p* < 0.001), but neither group effect (*F* = 1.332, *p* = 0.273) nor group × ROI effect (*F* = 0.483, *p* = 0.897). *Post hoc* analyses on ROI effect revealed that pain-related GBO increase was significantly higher in frontal sites than central (*p* = 0.001) and parietal (*p* < 0.001) sites and a higher tendency in frontal sites than temporal sites (*p* = 0.009) after Bonferroni correction (Figure 5). These results indicated that pain-related GBO increase was mostly significant in frontal region in all three groups. However, pain-related changes of frontal GBOs showed no significant correlation with pain tolerance measurement (total ET: *r* = 0.077, *p* = 0.591; ET to significant pain: *r* = 0.103, *p* = 0.473; perceived pain intensity: *r* = 0.143, *p* = 0.313; perceived unpleasantness: *r* = 0.086, *p* = 0.546).

**FIGURE 6:**
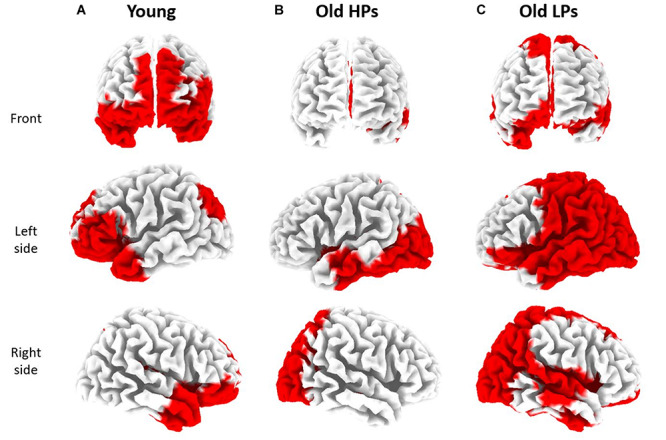
sLORETA based source localization on significant state differences (RS vs. SP) of gamma-band oscillations (GBOs) in three groups. Significantly increased GBOs were observed in frontal and occipital region for younger participants **(A)**, in parietal and occipital region for elderly high performers [HPs, **(B)**], and in frontal, parietal, and occipital regions for elderly low performers [LPs, **(C)**].

Furthermore, source localization based on sLORETA presented different patterns on pain-related GBO increase among three groups (Figure 6). Compared to RS, significantly increased GBOs induced by pain were located in frontal region for the younger participants, whereas in parietal and occipital regions for the elderly HPs. Elderly LPs, however, activated GBO power in more spread regions than the other two groups, including frontal, central, parietal, and occipital regions.

## Discussion

This study investigated the role of frontal functions in pain tolerance during aging. We observed that, in elderly subjects, better preservation of executive functions was associated with better pain-tolerance behavior. That is, elderly participants with higher cognitive performances tolerated pain longer than did those with lower cognitive performances. Increase of GBOs during pain tolerance in the frontal regions has been observed in all three groups, and elderly LPs showed GBOs increase in a more spread pattern compared with the other two groups. These results again were supportive to the age-related difference on pain tolerance and, more importantly, demonstrated the individual variability in pain tolerance within elderly population associated with functioning deterioration of the frontal cortex, at both behavioral and brain activity levels.

We observed that ET to pain differed between elderly HPs and LPs, who were assigned to these groups based on their performances in tests assessing executive functions. These results are consistent with previous evidence of a link between executive functions [i.e., inhibition (Oosterman et al., 2010) and working memory (Buhle and Wager, 2010; Legrain et al., 2013)] and pain tolerance. In the present study, executive functions were assessed using five tests. The use of multiple tests to evaluate executive functions and the higher threshold for defining low performance allowed a more sensitive assessment of executive functions than in previous studies. Our findings showed that healthy elderly individuals with preserved executive functions tolerate more efficiently sustained pain than healthy elderly persons with decline in executive functions, as revealed by longer ETs to pain. We also observed that, although ETs to pain were lower in elderly LPs than in elderly HPs and younger subjects, subjective judgments of pain intensity perception did not differ among these three groups. This may simply be due to the fact that the tolerance threshold for sustained pain is lower for HPs than for younger subjects and lower for LPs than for HPs, but perceived pain at the tolerance threshold is the same in all three groups.

Our spectral power analysis provided supportive evidence of cortical activities to the behavioral results, which identified significant age-related difference as well as interindividual difference within elderly subjects. We observed significant group effect on theta and low gamma power, which may indicate that age-related difference on pain-free state brain activity could affect pain tolerance. This result was partly in accordance with a recent research which observed increased connectivity between the two frequencies activities during resting state in chronic pain patients (Ta Dinh et al., 2019). Furthermore, we detected significant state effect on GBOs in the frontal sites in all three groups. Gamma-band oscillations have been believed to reflect the synchronous activity of large ensembles of rhythmically firing neurons and functionally have been suggested to reflect the local encoding of sensory, motor, or cognitive information. Tolerating pain involves complex encoding processing including both bottom-up–mediated factors such as stimulus intensity (e.g., encoding pain) and top-down–mediated factors such as cognitive control (e.g., inhibit motor reflex of removing hand from a heat box). Our study found no direct relationship between pain-related change of GBOs and pain behavioral measures. This result could be due to the fact that our pain behavioral measures were assessed at only one time point (i.e., at the end of tonic pain test), whereas change of GBOs was a continuous process. A real-time correlation could be more appropriate to reveal the relationship behavioral data and electrophysiological data in the tonic pain test.

Our findings of source analysis that GBOs induced by tonic pain were localized in the frontal regions in all three groups were consistent with findings in tonic pain models (Peng et al., 2014; Schulz et al., 2015). Frontal gamma activity during tonic pain may suggest that the encoding of tonic pain involves less sensory processes, but more affective processes and modulation processes. We believed that our tonic pain model adequately induced top-down pain modulation brain activity sustained by frontal functioning for all subjects. Nevertheless, the elderly LPs showed more spread GBO increase than the other two groups but still poor in tolerance pain. As the PFC is a crucial structure involved in top-down modulation of pain (Lorenz et al., 2003; Wiech et al., 2008; Moont et al., 2012; Bushnell et al., 2013), our result may reveal functional deficiency of cognitive or emotional pain modulation in elderly LPs. Given that experimental phasic pain was repeatedly shown to enhance gamma oscillations in the primary somatosensory and insular cortex, with gamma power correlating with subjective pain perception (Gross et al., 2007; Schulz et al., 2012; Zhang et al., 2012; Liberati et al., 2018b; Hu and Iannetti, 2019), and the bottom-up rather than the top-down modulations of phasic pain were encoded by GBOs (Tiemann et al., 2015), our results could reveal that, during tonic pain, the elderly LPs underwent more spread pain encoding processes in central and parietal regions and resulting in “intolerable pain.” Further studies are encouraged to directly contrast bottom-up and top-down modulations of tonic pain in elderly HPs and LPs to uncover the underlying mechanism on individual difference on pain tolerance varying with frontal functions.

The present study has some limitations. The most important would be the potential confound of gamma activity changes by muscle activity. As discussed by previous studies (Muthukumaraswamy, 2013), the analysis of high-frequency neural activity with EEG is strongly challenged by electromyogenic artifacts. In pain-tolerating task, muscle artifacts induced by facial expressions of pain may interfere with the signals recorded on frontal electrodes. We had conducted a few suggested analyses (Hipp and Siegel, 2013; Muthukumaraswamy, 2013) (e.g., blind source separation, saccadic movement detection by EOG electrodes, and source analysis) with the aim of reducing muscular artifact; however, to the best of our knowledge, no method can guarantee data free of high-frequency artifacts (Muthukumaraswamy, 2013). In future studies on pain-related GBOs, other approaches are encouraged, such as Laplacian montages, additional electromyographic electrodes over key muscle groups, which can be extremely informative in artifact identification and elimination. Another limitation of our study was the low spatial resolution of source localization due to limited EEG electrodes (i.e., 32 electrodes); therefore, more detailed and accurate distribution of increased GBOs induced by tonic pain is to be verified by future studies.

In summary, we observed that tonic pain processing was closely related to frontal functions, as shown by performances on neuropsychological tests (executive functions) and brain oscillations. In elderly subjects, preserved executive functions and effective frontal activations (top-down modulation) were associated with more efficient behavioral pain-tolerance abilities. These findings may provide a better comprehension of geriatric pain mechanisms and may assist in the development of more effective treatment plans for older patients with chronic pain.

## Data Availability Statement

The datasets generated for this study are available on request to the corresponding author.

## Ethics Statement

The studies involving human participants were reviewed and approved by the Ethics Committee of University of Strasbourg. The patients/participants provided their written informed consent to participate in this study.

## Author Contributions

SZ, OD, and AD: design. TP: experimental device. SZ: data collection. SZ, XB, and AD: analysis. SZ, SL, and AD: writing.

## Conflict of Interest

The authors declare that the research was conducted in the absence of any commercial or financial relationships that could be construed as a potential conflict of interest.

## Footnotes

1 www.statsoft.com

## Supplementary Material

FIGURE S1 Spectral power maps of alpha-band oscillations during resting-state **(A)** and significant tonic heat pain **(B)**. Statistical significance maps by paired *t*-test for each electrode and each group **(C)**, and significance maps after FDR correction **(D)**.

FIGURE S2 Spectral power maps of theta-band oscillations during resting-state **(A)** and significant tonic heat pain **(B)**. Statistical significance maps by paired *t*-test for each electrode and each group **(C)**, and significance maps after FDR correction **(D)**.
