# Supplementary figures and images for: Involvement of Frontal Functions in Pain Tolerance in Aging: Evidence From Neuropsychological Assessments and Gamma-Band Oscillations
Source: Front Aging Neurosci. 2020 May 27;12:131. doi: 10.3389/fnagi.2020.00131 (PMC7266988; doi:10.3389/fnagi.2020.00131)

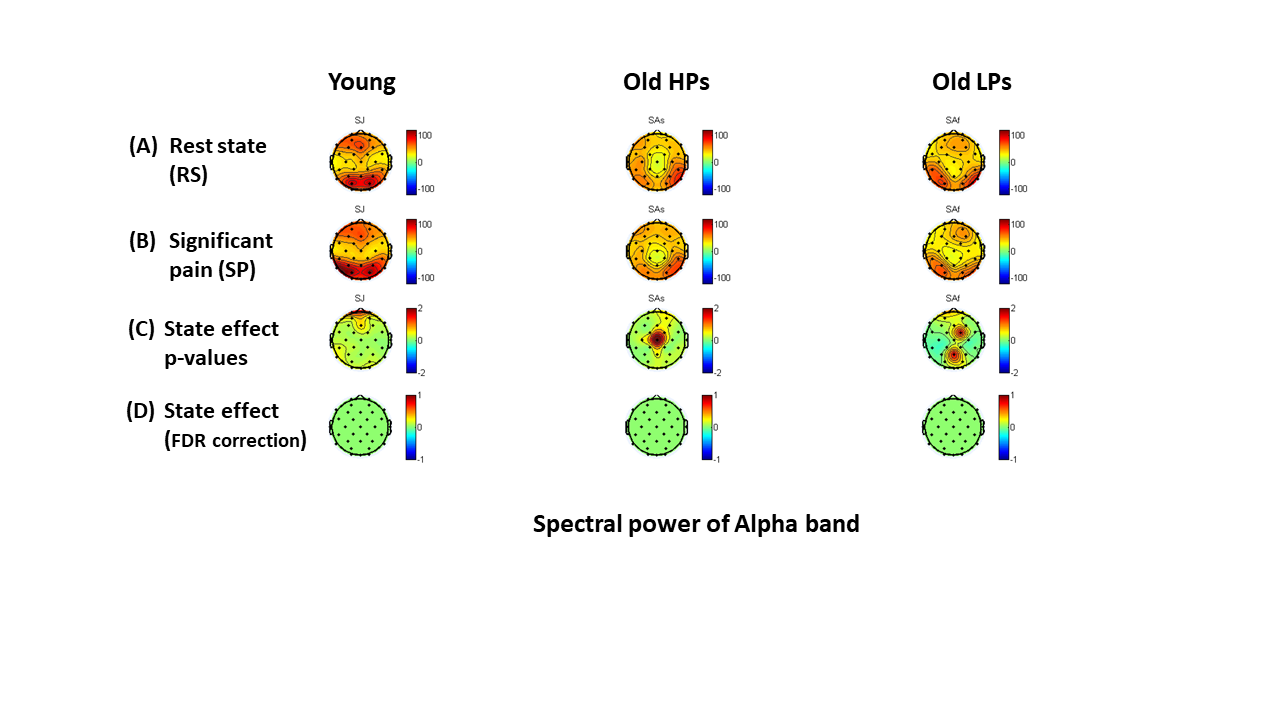

Supplement: FIGURE S1 — Spectral power maps of alpha-band oscillations during resting-state (A) and significant tonic heat pain (B). Statistical significance maps by paired t-test for each electrode and each group (C), and significance maps after FDR correction (D). [file Image_1.TIF]

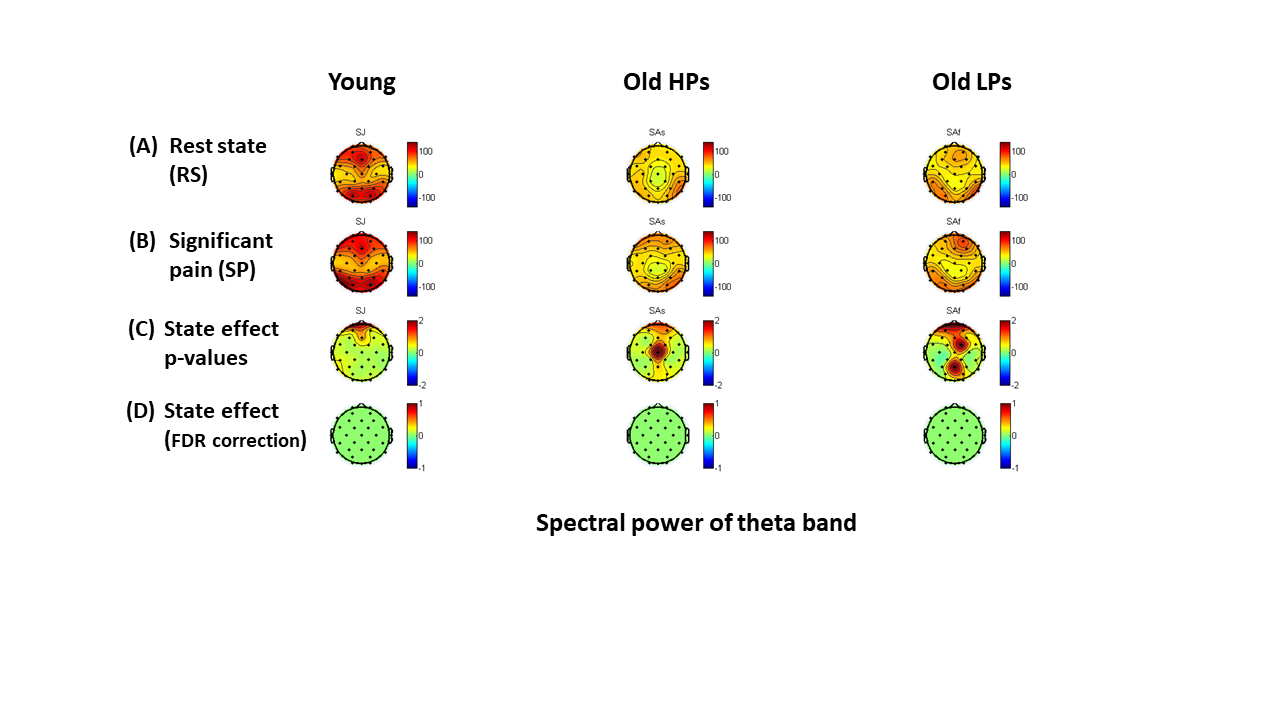

Supplement: FIGURE S2 — Spectral power maps of theta-band oscillations during resting-state (A) and significant tonic heat pain (B). Statistical significance maps by paired t-test for each electrode and each group (C), and significance maps after FDR correction (D). [file Image_2.TIF]
